# Clinical Evaluation of Specific Oral Manifestations in Pediatric Patients with Ascertained versus Potential Coeliac Disease: A Cross-Sectional Study

**DOI:** 10.1155/2014/934159

**Published:** 2014-08-13

**Authors:** Ennio Bramanti, Marco Cicciù, Giada Matacena, Stefano Costa, Giuseppe Magazzù

**Affiliations:** ^1^Resident Department of Clinical and Experimental Medicine and Stomatology, University of Messina, 98100 Messina, Italy; ^2^Department of Human Pathology, School of Dentistry, University of Messina, Via Consolare Valeria, 98100 Messina, Italy; ^3^Department of Pediatric, Pediatric Gastroenterology and Cystic Fibrosis Unit, University of Messina, 98100 Messina, Italy

## Abstract

Patients involved on coeliac disease (CD) have atypical symptoms and often remain undiagnosed. Specific oral manifestations are effective risk indicators of CD and for this reason an early diagnosis with a consequent better prognosis can be performed by the dentist. There are not researches analysing the frequency of these oral manifestations in potential coeliac patients. The aim of this study is to investigate the oral hard and soft tissue lesions in potential and ascertained coeliac children in comparison with healthy controls. 50 ascertained children, 21 potential coeliac patients, and 54 controls were recruited and the oral examination was performed. The overall oral lesions were more frequently present in CD patients than in controls. The prevalence of oral soft tissue lesions was 62% in ascertained coeliac, 76.2% in potential coeliac patients, and 12.96% in controls (*P* < 0.05). Clinical dental delayed eruption was observed in 38% of the ascertained coeliac and 42.5% of the potential coeliac versus 11.11% of the controls (*P* < 0.05). The prevalence of specific enamel defects (SED) was 48% in ascertained coeliac and 19% in potential coeliac versus 0% in controls (*P* < 0.05; OR = 3.923). The SED seem to be genetically related to the histological damage and villous atrophy.

## 1. Introduction

Coeliac disease (CD), one of the most frequent chronic diseases among Caucasians, is an immune-mediated enteropathy that affects genetically susceptible subjects following exposure to gluten in the diet. Gluten is a proline-rich and glutamine-rich protein present in wheat (gliadin), rye (secalin), and barley (hordein), which is able to enhance an immune-mediated inflammation in intestinal mucosa and outside the gut [1].

Coeliac disease develops as a consequence of the association between this environmental trigger and a genetically predisposed host (HLA-DQ2/DQ8 genes), with the possible concurrence of other environmental cofactors.

This condition leads the patients to an inflammatory enteropathy, with villous atrophy of the intestinal mucosa, crypt hyperplasia, and an inflammatory infiltrate in the adjacent connective tissue, associated with an increase of intraepithelial lymphocytes [2].

A considerable increase in the prevalence of CD has been recorded, ranging from 1 : 85 to 1 : 300 according to the considered geographic area [3], probably due to the wheat-consuming affluent societies of the western world and to the improved reliability of serological tests (measurement of antitransglutaminase antibodies tTG and antiendomysium antibodies EMA) recorded in the recent decades.

*Potential coeliac disease* is diagnosed in patients who report positive coeliac-related antibodies but with normal mucosa at the jejunal biopsy. Although these patients are at risk for developing a typical CD enteropathy later in life, there is no evidence to keep managing them with a gluten-free diet or rigorous followup until unequivocal mucosal flattening is recorded [1].

Patients with* ascertained coeliac disease* show positive serological patterns with damage to the intestinal mucosal architecture. For them the unique proven treatment is rigorous and life-long adherence to a gluten-free diet [4].

Coeliac disease is called the “clinical chameleon” because it may present with a large variety of nonspecific signs and symptoms. It is important to diagnose CD not only in children with classical gastrointestinal symptoms but also in children with a less clear clinical portrait [5, 6].

The CD with* nonclassic symptoms* manifests with unusual intestinal complaints (e.g., abdominal pain, gassiness/increased flatulence, nausea, vomiting, bloating, and constipation) or extraintestinal manifestations: short stature, anemia not responsive to iron therapy, hepatic dysfunction, dermatitis herpetiformis, xerosis, cutaneous vesicles, arthralgia, and myalgia [7, 8].

Nowadays it is widely recognized that, among these atypical signs of CD, there are some oral manifestations which are strictly related to ascertained coeliac disease: dental enamel defects, recurrent aphthous stomatitis, delayed tooth eruption, multiple caries, angular cheilitis, atrophic glossitis, dry mouth, burning tongue [9–15].

For this reason oral pathologists and dentists have a key role for early diagnosis of CD and its secondary prevention [16].

Already in 1986, Aine has first described in children dental enamel defects that are exclusively related to coeliac disease [17]. These* specific enamel defects* have to be symmetrically and chronologically detectable in all four sections of the dentition. Other enamel defects (discolorations, hypoplasias, or opacities) that are not symmetrical and chronological and not involved in the same teeth in both hemiarches are considered* unspecific*. Moreover Aine classified the specific enamel defects in grades I–IV according to the severity of their clinical aspect.

Despite the established significant link between oral disease and CD, scientific literature has no researches that analyze or correlate the oral pathological manifestations with the potential coeliac disease in patients with nonatrophic intestinal lesions.

Another aim of the present study is to highlight how the specific enamel defects described by Aine, evidently typical of ascertained CD, are associated with the histopathological jejunal damage or to the serological autoantibodies action.

This study has the main purpose to evaluate the prevalence of oral manifestations (i.e., specific and unspecific enamel defects, recurrent aphthous stomatitis, delayed dental eruption, angular cheilitis, geographic tongue, atrophic glossitis, and multiple caries) in potential nonatrophic coeliac patients by comparing them with ascertained coeliac patients with atrophic intestinal mucosa and also with healthy subjects.

## 2. Materials and Methods

From December 2012 to July 2013 a total of 116 subjects consecutively referred to the Department of Pediatric Gastroenterology and Cystic Fibrosis of “AOU Gaetano Martino” Hospital in Messina, with the suspected diagnosis of CD due to the presence of gastrointestinal symptoms, CD familiarity, or screening. They underwent the first specialist paediatric visit for the diagnostic assessment of CD.

ESPGHAN guidelines have been used for the diagnosis of coeliac disease [18]. It was based on the positivity of CD-related serological tests (antitransglutaminase antibodies tTG, antiendomysium antibodies EMA, antigliadin antibodies AGA, and the typical HLA-predisposing genotype) followed by histological confirmation on duodenal biopsy.

The Oberhuber classification [19] was used to grade the severity of histopathological lesions and to classify the CD.

In 103 patients coeliac disease was diagnosed, while CD was excluded in the other 13 subjects. Alternative hypothesis for their symptoms was viral infections, bacterial infections, or immunological reactions.

Among CD subjects, only patients aged between 2 to 16 years old were included in this study.

So a sample of 83 little patients who completed a questionnaire remained, with parents' help, about their medical and dental history. Questions about comorbidities use of medication or nutritional supplements, fluoride, and alimentary habits were included.

Patients who reported at medical anamnesis the following diseases, which often present oral manifestations for other etiological reasons, in order to avoid bias for sampling and to get a direct correlation between CD and oral manifestations, constituted the exclusion criteria:

- Behcet's syndrome,
- diabetes mellitus,
- immunodeficiency,
- Reiter's syndrome,
- Crohn's disease,
- ulcerative colitis,
- deficiency of A, B12, C, or D vitamins
- lichen planus,
- syphilis,
- endocrine pathologies.

Other exclusion criteria were highlighted by patients' responses to the questionnaire and were represented by the genetic heredity to amelogenesis imperfecta and an excessive intake of fluoride or tetracycline because both represented verified etiological factors of dental enamel defects.

Furthermore CD patients, who previously made a gluten-free diet for a period of one year or more, were excluded from this study in order to avoid obtaining results distorted by diet therapeutic effects.

Following this sample's selection, 71 patients were elected to participate in this cross-sectional study. Everyone was available to take part in it and parents' subjects provided written informed consent.

These 71 enrolled patients were divided into two groups, according to the histopathological diagnosis they received:

- *A Group*: 50 ascertained coeliac patients with 2 or 3 histotype according* Oberhuber*,
- *B Group*: 21 potential coeliac patients with 0 or 1 histotype according* Oberhuber*.

At the same department, when children and their parents received the confirmed diagnosis, every patient was subjected to a specialist dental visit performed by the same blind clinician, who did not know patients' diagnosis and treatment.

Fifty-four healthy subjects who were age-/sex-matched (22 M, 32 F, age range: 2–16 years, average: 8.8 ± 2.9, median: 9) and living in the same geographical area as that of the CD group were enrolled as controls.

Pediatric controls were recruited among otherwise healthy patients consecutively referred to the Pediatric Dental Unit of the Department of Odontostomatology at Messina University for restorative treatments.

These subjects did not refer any diseases, had no family history of CD, and showed normal growth (weight/height ratio between 25th and 75th centiles).

Informed consent was obtained for all control subjects of the study.

These healthy patients belonged to the C Group.

For all 125 children of this study, the same blind dentist evaluated the following intra- and extraoral clinical manifestations that in literature have been analyzed only in ascertained coeliac patients [14–17, 20]:

- specific and unspecific enamel defects (SED-unSED),
- dental delayed eruption (DDE),
- recurrent aphthous stomatitis (RAS),
- geographic tongue (GT),
- burning tongue (BT),
- atrophic glossitis (AG),
- angular cheilitis (AC),
- dental caries.

Any hard and soft tissue oral lesion was clinically recorded, described, classified, and finally photographed by the blind dentist.

Specific enamel defects (SED) affecting deciduous and permanent teeth were graded I–IV (Table 1) according to Aine's classification [17].

The assessment of RAS included recurrent aphthous ulcers clinically observed by the investigator and also recurrent ulcerative lesions, noted by parents or patients, or reported in hospital clinical records, with clinical features of RAS.

To evaluate DDE we used the conventional eruption tables for the Caucasian population [21], by considering delayed eruption as when the teeth were not in arch after their normal age of eruption, with 6-month range.

Dental caries were recorded as DMFT/dmft indices (decayed, missed, and filled permanent/deciduous teeth), recommended by the World Health Organization [22].

Results were finally recorded and compared between A and B groups through a* statistical analysis* performed using GraphPad Prism 5. Categorical variables were expressed as numbers and percentages.

These variables were analyzed by cross-tabulations using *χ*_2_ test or Fisher's exact test, as appropriate. *P* value <0.05 was considered the level of statistical significance.

Institutional Ethical Committee Board of the University Hospital of Messina approved this study.

## 3. Results

A total sample of one hundred and twenty-five children was examined: 50 ascertained coeliac patients belonged to the A Group, 21 potential coeliac patients belonged to the B Group, and 54 control subjects belonged to the C Group.

The male : female ratio was 22 : 28 in A Group, 6 : 15 in B Group, and 22 : 32 in C Group.

The mean age was, respectively, 7.5 ± 4.4, 6.9 ± 3.9, and 8.8 ± 2.9 years old.

Specifically, in A Group histopathological results reported 33 patients (66%) with 3C-histological type, 8 patients (16%) with 3B + 3C, 3 patients (6%) with 3A + 3B, 2 patients (4%) with 3A, and 4 patients (8%) with 2-type.

In B Group the same Oberhuber classification showed 11 patients (52.4%) with 1-histological type, while 10 children (47.6%) did not present any damage to the intestinal mucosa, but only positive serological markers for CD.

Instead in C Group, no patient suffered from clinical symptoms that might cause malabsorption processes, including diarrhoea and vomiting neither from any gastrointestinal pathology.

In regard to medical history, it was to underline only the frequent anemia not responsive to iron therapy registered in coeliac groups (34% A Group and 57% B Group), which considerably decreased in the control group (18.5%).

## 4. Oral Soft Tissue Lesions

**Figure 1:**
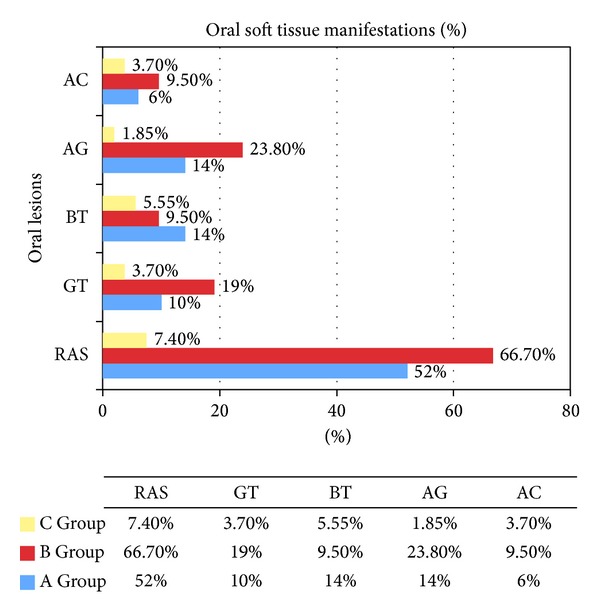
Oral soft tissue manifestations in ascertained and potential coeliac patients and in healthy subjects.

Figure 1 and Table 1 summarized the main findings of the specific oral soft tissue lesions in all CD patients and controls.

The* recurrent aphthous stomatitis* appeared small, round, or ovoid shaped with circumscribed margins, erythematous haloes, and yellow but more often gray floors.

*RAS* was found in 26/50 (52%) ascertained coeliac patients, 14/21 (66.7%) potential coeliac patients, and 4/54 (7.4%) controls. In CD patients, RAS were directly observed by the clinician during the dental visit in 38 cases and simply referred by parents in two cases.

Furthermore it was found that* RAS* was more frequent in silent coeliac patients, who did not report any gastrointestinal symptoms before the diagnosis of CD.

**Figure 2:**
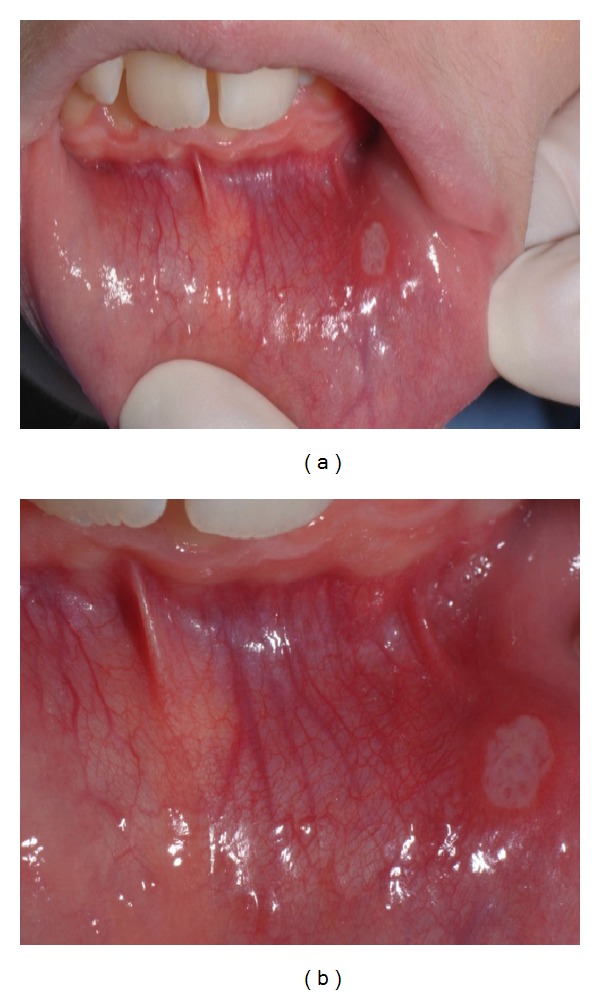
Mucosal aphthous stomatitis in an ascertained coeliac patient (A Group).

**Figure 3:**
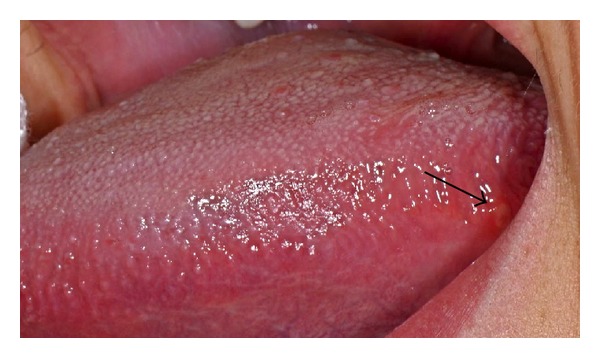
Unilateral aphthous ulcer at lingual margin in a potential coeliac patient (B Group).

The most common sites of* RAS* were the labial mucosa and tongue's lateral margins (Figures 2(a), 2(b), and 3).

The* geographic tongue* appeared as an atrophic surface without papillae, which was bordered by a slightly raised, white, yellow, or grey peripheral zone. This condition often affected only part of the tongue, especially upon its tip. It was found in 5/50 (10%) ascertained coeliac patients, 4/21 (19%) potential coeliac patients, and 2/54 (3.7%) control subjects.

*Burning tongue* was recorded as a mix of subjective burning sensations associated with objective signs of erythema and edema of papillae on the tip of the tongue not linked to any type of physical trauma. It was registered in 7/50 (14%) patients of the A Group, 2/21 (9.5%) patients of the B Group, and 3/54 (5.55%) patients of the C Group. However this clinical sign seemed to be particularly linked to patients' anaemic state.

In* atrophic glossitis* tongue's surface appeared smooth and erythematous and patients referred difficulty with chewing, swallowing, or, less time, speaking.

This condition was noted in 7/50 (14%) ascertained coeliac patients and 5/21 (23.8%) potential coeliac patients versus 1 out of 54 (1.85%) controls.

**Figure 4:**
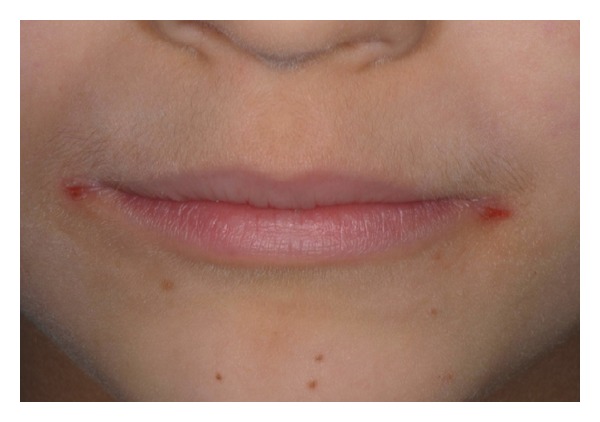
Angular cheilitis of minor size in a potential coeliac patient (B Group).

The* angular cheilitis* was of minor and mild size and was characterized by diffuse redness with an eroded, fissured, ulcerated, or encrusted surface (Figure 4).

This extraoral condition was recorded in 3/50 (6%) patients of the A Group and 2/21 (9.5%) patients of the B Group versus 2/54 (3.7%) patients of the control Group.

Young patients referred their spontaneous onset without physical trauma by tongue or nail scratching and with frequent symptoms of burning, tenderness, or pain.

## 5. Oral Hard Tissue Lesions

Consequently we evaluated oral hard tissue lesions and analyzed the prevalence of caries, dental delayed eruption, and specific and unspecific enamel defects in ascertained and potential coeliac patients and controls.

The decayed, missed, and filled teeth* indices* fell within the standard range and they were recorded for decidual (dmft) and permanent (DMFT) teeth. In A Group dmft was 1.07 ± 1.63 and DMFT 2.52 ± 3.22; in B Group dmft was 1.6 ± 2.67 and DMFT 1.57 ± 1.87; in the control Group dmft was 1.86 ± 1.98 and DMFT 2.41 ± 1.63.

*Clinical dental delayed eruption* was observed in 19 ascertained coeliac patients (38%) with an average value of 1.4 years of delay and in 9 potential coeliac patients (42.8%) with an average of 1.7 years of delay. Among the control group, 6 healthy subjects (11.1%) presented a delay of tooth eruption which was not clinically significant because less than one year of average.

The presence of* unspecific enamel defects* (UnSED) was recorded in 6/50 (12%) ascertained coeliac patients* versus* 1 out of 21 (4.7%) potential coeliac subjects.

There was not a statistically significant difference between the two CD groups (two-sided Fisher's exact test—*P* value = 0.6654).

Also 3/54 (5.55%) healthy subjects presented unspecific enamel defects, which were neither symmetrical nor chronological in hemiarches.

**Figure 5:**
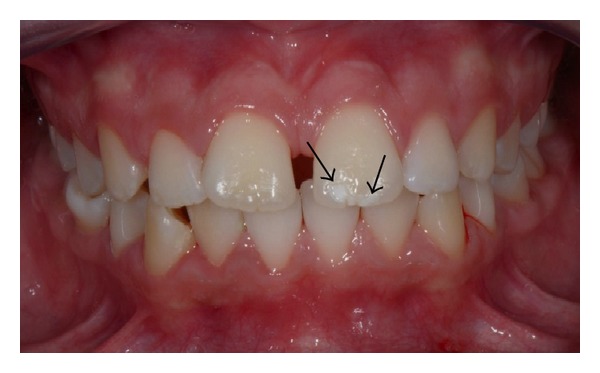
Unspecific enamel defects (white spots) upon the incisal edge of the upper left central incisor in ascertained coeliac patient (A Group).

Figure 5 showed an unspecific colour enamel defect observed in an ascertained coeliac patient who was included in the study.

*Specific enamel defects* (SED) were symmetrical and chronological colour or structural defects which involved the same teeth in both hemiarches considered.

They were completely absent in the healthy controls (0/54), while they have been registered in different amounts between A and B Groups.

A total of 24/50 (48%) ascertained coeliac patients showed* SED* against 4/21 (19%) potential coeliac subjects with a statistical significant difference [*P* = 0.0328; OR = 3.923 (95% CI = 1.155 : 13.32)].

**Figure 6:**
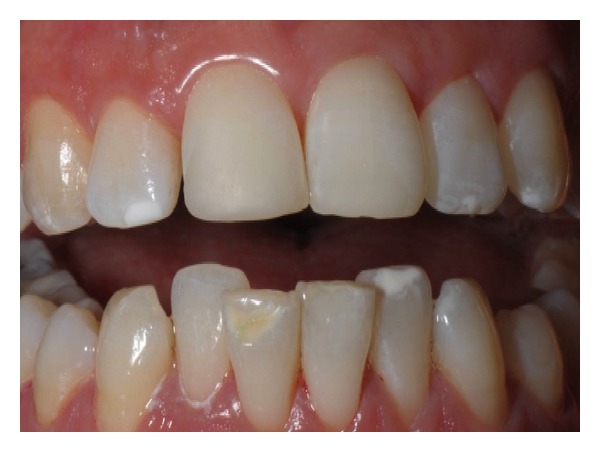
Grade II specific enamel defects in a potential coeliac patient (B Group). Symmetrical and chronological position on the following dental elements: 12-13-22-23-32-41.

**Figure 7:**
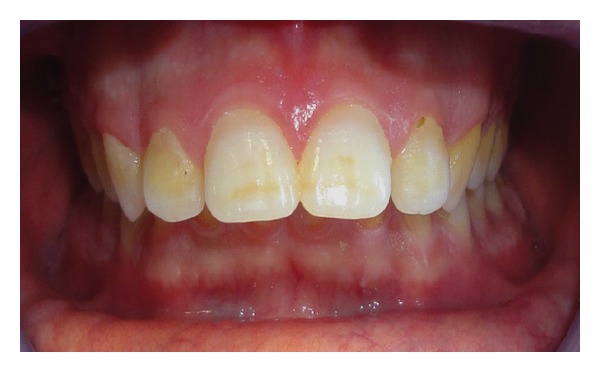
Grade III specific enamel defects in an ascertained coeliac patient (A Group). Symmetrical and chronological position on the following dental elements: 11-12-13-14-21-22-23-24.

SED severity was evaluated according to Aine's classification [17] and reported a higher frequency of mild I and II grades: 15 ascertained (62.5%) and 2 potential patients (50%) had grade I SED; grade II was found in 7 children of A Group (29.17%) and 2 children (50%) of B Group (Figure 6), while grade III SED characterized only 2 ascertained coeliac subjects (8.33%) (Figure 7).

These adamantine defects predominantly presented cream or grey discolorations with rough enamel surface partially without glaze and some horizontal grooves.

Recorded SED were easily recognizable but did not radically change the shape of the tooth, so the functional aspects were not compromised.

SED were found more frequently in permanent teeth and mainly affected the premolars and the frontal group.

**Figure 8:**
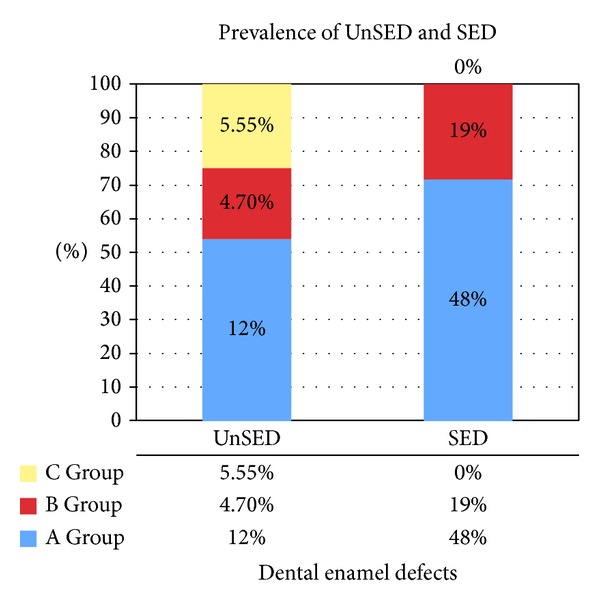
Prevalence of unspecific (UnSED) and specific (SED) enamel defects in all groups.

The statistical prevalence of UnSED and SED in the three sample groups of this study is reported in Figure 8.

## 6. Discussion

Recent epidemiological studies demonstrated a prevalence of CD approaching 1% in the general population [23, 24]. The advent of serology at the end of the 1980s signed a significant trend of an increased CD diagnostic rate, a progressive lowering of patients' age at diagnosis, and a reduction of people with overt malabsorption, which started in Italy and was confirmed in USA [25].

Although several researches have shown the presence of oral mucosal lesions in CD, our study reports the first evaluation of the risk and the prevalence of such lesions in potential coeliac patients, whose oral manifestations had never been analyzed.

Coeliac disease has a wide clinical heterogeneity: patients can range from asymptomatic to severely symptomatic. For classifying the possible forms of clinical presentations, terms such as silent, atypical, and typical can clearly and simply characterize the clinical presentation.

The factors related to the type and the severity of clinical presentation still remain unknown. Researchers have shown that neither the extent of duodenal villous atrophy nor the degree of visible enteropathy assessed by capsule endoscopy correlate with presentation [26].

Nowadays there has been a remarkable change in clinical presentation of coeliac disease, with almost 50% of patients with newly diagnosed CD who do not present with gastrointestinal symptoms, thus making diagnosis complex.

Studies have calculated that the burden of undetected CD is very high and the ratio between diagnosed and undiagnosed patients even is 1 : 7 [27].

Thus, in order to recognize the greatest number of “atypical” or “silent” CD patients and prevent complications, clinicians must investigate the known “at-risk subjects” (those with chronic anaemia, hypertransaminasemia, osteoporosis, type 1 diabetes, autoimmune thyroid disorders, and dermatitis herpetiformis) and also examine other possible risk factors that remain partially unclear.

Some specific oral lesions seem to represent risk indicator of CD [6, 7].

Although the proximal part of the intestinal mucosa is the main gut's site involved in CD, it has been proved that gluten-driven T-cell activation is present in the whole gastrointestinal tract, including the mouth. Several studies have confirmed the frequent occurrence of oral lesions related to both mineralized and soft tissues in patients with coeliac disease, especially in children and young adults [11–16]. The knowledge and recognition of these oral signs could give a useful diagnostic contribution to the CD.

The results of this cross-sectional study compared the oral clinical lesions in pediatric patients with potential and ascertained coeliac disease and also in healthy subjects with the aim of verifying if there is an etiopathogenetic link between villous intestinal atrophy and oral manifestations.

The whole oral lesions considered in this research were significantly more frequent in patients with coeliac disease compared to healthy patients.

The unique exception was represented by the dmft/DMFT scores, which did not greatly vary from the standard range for all three groups, despite a slight overestimation for the control group that referred to our clinician at Pediatric Dental Unite for restorative needs.

For this reason, results of our study were discordant with other researches that indicated an increased caries incidence in coeliac patients [28].

Major percentages of oral soft tissue lesions were recorded among potential coeliac patients (75% *n* = 18), while oral hard tissue lesions involved more frequently ascertained coeliac subjects (68% *n* = 34). However, the histopathologic severity of intestinal mucosa damage was neither correlated nor proportional to the severity of oral tissue lesions.

As regards the clinical manifestations of CD, “typical” gastrointestinal symptoms were more often observed in ascertained coeliac children (32/50 cases, 64%) than in potential ones (8/21 cases, 38.1%). Cases of oral soft and hard tissue lesions in patients without any signs and symptoms potentially related to CD and diagnosed during familiar CD screening should be considered as patients with “atypical” and not with “silent” CD.

An “atypical” or “silent” CD was the clinical presentation of 18 (36%) coeliac patients and 13 (61.9%) potential ones.

Among the latter, 14 (77.78%) ascertained coeliac patients and 10 (76.92%) potential coeliac subjects presented some specific oral signs. These high percentages have clearly underlined that the dentist might hold a key role in early CD diagnosis and secondary prevention, especially in asymptomatic and atypical patients. The dentist could be the first specialist to get the diagnostic doubt of apparently unsuspected coeliac disease from the survey of these clinical features.

Among our patients, dental enamel defects were more frequently observed in coeliac children with “typical” gastrointestinal symptoms (61%), while oral soft tissue lesions were noted in more patients with “atypical” and “silent” CD (54%).

The soft tissue evaluation highlighted mucosal and cutaneous lesions found in about 76.2% of the potential coeliac patients (*n* = 16) and in 62% (*n* = 31) of the ascertained coeliac children, against 12.96% (*n* = 7) of the controls.

It was also interesting that the 12.76% of these coeliac patients (6/47) presented only one specific soft tissue lesion. The contemporary presence of two or more oral mucosal and cutaneous clinical signs was very frequent.

However, the most important finding of the present study was related to the oral hard tissue investigation: in fact we found systematic, chronological, and symmetrical enamel defects in 48% of the ascertained coeliac patients, with an OR > 3 in comparison with potential coeliac patients. They were easily recognizable structural and colour defects of the enamel surface of low and moderate grades according to Aine [17].

Our data about SED degree and position were in agreement with other studies performed in Italy [11–13, 29, 30], but our research reported a higher frequency which probably depended on Mediterranean geographic area, environmental, dietetic, and also genetic factors.

The analysis of SED in potential coeliac patients and the results obtained from this study could indicate an etiopathogenetic hypothesis of SED.

In fact the mechanism of the development of dental enamel hypoplasia caused by gluten in patients with coeliac disease is still unclear. There are three hypotheses.

Nikiforuk and Fraser indicated that a low serum calcium concentration during enamel formation is a specific cause of enamel hypoplasia [31].

The study of Mariani et al. [29] suggested that the HLA-DR3 antigen significantly increases the risk of dental lesions, indicating a genetic cause. Another recent publication by Erriu et al. underlined how the presence of the HLA-DQB1*02 allele can influence the development of oral signs in a dose-dependent manner and the HLA haplotype connected to oral signs could have a fundamental role for the diagnosis of atypical forms of CD [32, 33].

Aine et al. [34] and Maki et al. [35] described the damage of the enamel organ as the consequence of an autoimmune response.

The results of this study excluded the first hypothesis because 11 ascertained coeliac patients and 2 potential coeliac children without malabsorption processes and with a normal serum calcium concentration presented SED.

A specific antigen described by Mariani et al. [29] in the second hypothesis might be the logic clarification for the fact that not all coeliac patients are affected by SED.

The hypothesis of an autoimmune response described by Maki et al. [35] is significantly congruous with the results obtained in our study.

The specific enamel defects seem to be related not only to the gluten-driven T-cell activation and serological autoantibodies action, but also to the histopathological jejunal damage and villous atrophic lesion.

The presence of SED in ascertained coeliac children with intestinal atrophic lesions was statistically significantly higher than in potential coeliac patients (48% against 19%; *P* = 0.0328; OR = 3.923).

A subject with ascertained coeliac disease had almost 4 times greater probability to have SED in comparison with a potential coeliac.

This significant difference between these two categories of coeliac patients indicated that the presence of positive serological markers of CD should not be sufficient to provoke SED.

In addition, all the 4 potential coeliac children of the study who presented SED had histopathological pattern of grade I according to* Oberhuber* with infiltrative lesion and increase of IELs (>25 IELs/100 enterocytes). No potential coeliac patient with grade 0 according to* Oberhuber* presented SED. Despite that, the severity of SED was not directly proportional to the grade of histopathological villous atrophic lesions.

The North American Society for Pediatric Gastroenterology, Hepatology, and Nutrition expressed a clinical practice guideline for the diagnosis and treatment of CD in children [2], in which dental enamel defects were specified as a symptom of coeliac disease. This guideline urgently advises the following procedure when dental enamel defects are noticed, because affected patients are considered “at-risk subjects.”

Finally, RAS associated with other oral soft tissue lesions, dental delayed eruption of more than one year, and SED advises for serological CD screening.

## 7. Conclusions

This study analyzed specific oral manifestations in pediatric potential coeliac patients in comparison with pediatric ascertained coeliac patients and pediatric healthy controls.

It showed a higher frequency of oral lesions in CD patients than in healthy subjects.

The oral soft tissue lesions were more frequent in potential coeliac patients, while oral hard tissue lesions affected a greater number of ascertained coeliac children.

Specific enamel defects could have an etiological link with the histopathological intestinal damage and villous atrophic lesion.

The preventive recognition of these specific oral lesions by the dentist should allow preventing the disease's manifestations on the mucosa. Moreover the patient health can be directed to better prognosis thanks to a suspected diagnosis of CD avoiding and anticipating the occurrence of gastrointestinal symptoms and more severe pathological injury.

## Figures and Tables

**Table 1:** Localization of the Oral soft tissue lesions and percentage on each group.

Oral manifestations	A Group	B Group	C Group
	% (*n*)	% (*n*)	% (*n*)
Recurrent aphotus stomatitis	52% (26)	66.7% (14)	7.4% (4)
Geographic tongue	10% (5)	19% (4)	3.7% (2)
Burning tongue	14% (7)	9.5% (2)	5.55% (3)
Atrophic glossitis	14% (7)	23.8% (5)	1.85% (1)
Angular cheilitis	6% (3)	9.5% (2)	3.7% (2)

## References

[1] Di Sabatino A, Corazza GR (2009). Coeliac disease. *The Lancet*.

[2] Hill ID, Dirks MH, Liptak GS (2005). Guideline for the diagnosis and treatment of celiac disease in children: recommendations of the North American society for pediatric gastroenterology, hepatology and nutrition. *Journal of Pediatric Gastroenterology and Nutrition*.

[3] Fasano A, Araya M, Bhatnagar S (2008). Federation of international societies of pediatric gastroenterology, hepatology, and nutrition consensus report on celiac disease. *Journal of Pediatric Gastroenterology and Nutrition*.

[4] Makharia GK, Catassi C, Goh KL, Mulder CJJ (2012). Celiac disease. *Gastroenterology Research and Practice*.

[5] Ferguson A, Arranz E, O'Mahony S (1993). Clinical and pathological spectrum of coeliac disease—active, silent, latent, potential. *Gut*.

[6] Dewar DH, Ciclitira PJ (2005). Clinical features and diagnosis of celiac disease. *Gastroenterology*.

[7] Admou B, Essaadouni L, Krati K (2012). Atypical celiac disease: from recognizing to managing. *Gastroenterology Research and Practice*.

[8] Caproni M, Bonciolini V, D’Errico A, Antiga E, Fabbri P (2012). Celiac disease and dermatologic manifestations: many skin clue to unfold gluten-sensitive enteropathy. *Gastroenterology Research and Practice*.

[9] Majorana A, Bardellini E, Ravelli A, Plebani A, Pol A, Campus G (2010). Implications of gluten exposure period, CD clinical forms, and HLA typing in the association between celiac disease and dental enamel defects in children. A case-control study. *International Journal of Paediatric Dentistry*.

[10] Seyhan M, Erdem T, Ertekin V, Selimoğlu MA (2007). The mucocutaneous manifestations associated with celiac disease in childhood and adolescence. *Pediatric Dermatology*.

[11] Campisi G, Di Liberto C, Iacono G (2007). Oral pathology in untreated coeliac disease. *Alimentary Pharmacology & Therapeutics*.

[12] Bucci P, Carile F, Sangianantoni A, D'Angiò F, Santarelli A, Muzio LL (2006). Oral aphthous ulcers and dental enamel defects in children with coeliac disease. *Acta Paediatrica*.

[13] Fasano A, Catassi C (2012). Clinical practice. Celiac disease. *The New England Journal of Medicine*.

[14] Acar S, Yetkiner AA, Ersin N, Oncag O, Aydogdu S, Arikan C (2012). Oral findings and salivary parameters in children with celiac disease: a preliminary study. *Medical Principles and Practice*.

[15] Wierink CD, van Diermen DE, Aartman IHA, Heymans HSA (2007). Dental enamel defects in children with coeliac disease. *International Journal of Paediatric Dentistry*.

[16] Rashid M, Zarkadas M, Anca A, Limeback H (2011). Oral manifestations of celiac disease: a clinical guide for dentists. *Journal of the Canadian Dental Association*.

[17] Aine L (1986). Dental enamel defects and dental maturity in children and adolescents with coeliac disease. *Proceedings of the Finnish Dental Society: Suomen Hammaslaakariseuran Toimituksia*.

[18] Husby S, Koletzko S, Korponay-Szabò IR (2012). European society for pediatric gastroenterology, hepatology, and nutrition guidelines for the diagnosis of coeliac disease. *Journal of Pediatric Gastroenterology and Nutrition*.

[19] Oberhuber G, Granditsch G, Vogelsang H (1999). The histopathology of coeliac disease: time for a standardized report scheme for pathologists. *European Journal of Gastroenterology and Hepatology*.

[20] Ferraz EG, de Jesus Campos E, Sarmento VA, Silva LR (2012). The oral manifestations of celiac disease: information for the pediatric dentist. *Pediatric Dentistry*.

[21] Nowak AJ (2001). *Oral Management of Pediatric Patients for Non-Dental Professionals, A Study Guide*.

[22] (1987). *Oral Health Surveys: Basic Methods*.

[23] Chin MW, Mallon DF, Cullen DJ, Olynyk JK, Mollison LC, Pearce CB (2009). Screening for coeliac disease using anti-tissue transglutaminase antibody assays, and prevalence of the disease in an Australian community. *Medical Journal of Australia*.

[24] Maki M, Mustalahti K, Kokken J (2003). Prevalence of coeliac disease among children in Finland. *The New England Journal of Medicine*.

[25] Murray JA, van Dyke C, Plevak MF, Dierkhising RA, Zinsmeister AR, Melton LJ (2003). Trends in the identification and clinical features of celiac disease in a North American community, 1950–2001. *Clinical Gastroenterology and Hepatology*.

[26] Brar P, Kwon GY, Egbuna II (2007). Lack of correlation of degree of villous atrophy with severity of clinical presentation of coeliac disease. *Digestive and Liver Disease*.

[27] Fasano A, Catassi C (2001). Current approaches to diagnosis and treatment of celiac disease: an evolving spectrum. *Gastroenterology*.

[28] Avşar A, Kalayci AG (2008). The presence and distribution of dental enamel defects and caries in children with celiac disease. *Turkish Journal of Pediatrics*.

[29] Mariani P, Mazzilli MC, Lionetti P (1994). Coeliac disease, enamel defects and HLA typing. *Acta Paediatrica, International Journal of Paediatrics*.

[30] Rea F, Serpico R, Pluvio R (1997). Dental enamel hypoplasia in a group of celiac disease patients: clinico-epidemiologic correlations. *Minerva Stomatologica*.

[31] Nikiforuk G, Fraser D (1981). The etiology of enamel hypoplasia: a unifying concept. *Journal of Pediatrics*.

[32] Erriu M, Sanna S, Nucaro A, Orrù G, Garau V, Montaldo C (2011). HLA-DQB1 haplotypes and their relation to oral signs linked to celiac disease diagnosis. *Open Dentistry Journal*.

[33] Jores R, Frau F, Cucca F (2007). HLA-DQB1*0201 homozygosis predisposes to severe intestinal damage in celiac disease. *Scandinavian Journal of Gastroenterology*.

[34] Aine L, Mäki M, Collin P, Keyriläinen O (1990). Dental enamel defects in coeliac disease. *Journal of Oral Pathology & Medicine*.

[35] Maki M, Aine L, Lipsanen V, Koskimies S (1991). Dental enamel defects in first-degree relatives of coeliac disease patients. *The Lancet*.

